# The Effects of Whole-Body Vibration Therapy in Weight-Bearing and Non-Weight-Bearing Positions for Upper and Lower Extremities on Balance and Function in Cerebral Palsy Children: A Randomized Controlled Trial

**DOI:** 10.7759/cureus.61404

**Published:** 2024-05-31

**Authors:** Syed Ali Hussain, Dr. Mohammad-Reza Hadian, Zainab Hassan, Azadeh Shadmehr, Saeed Talebian, Mubin Mustafa Kiyani, S. Mohsen Mir

**Affiliations:** 1 School of Rehabilitation, Tehran University of Medical Sciences, Tehran, IRN; 2 Brain and Spinal Injury Research Center, Institute of Neuroscience, Tehran, IRN; 3 Shifa College of Medical Technology, Shifa Tameer e Millat University, Islamabad, PAK

**Keywords:** whole-body vibration, weight bearing and non-weight bearing, motor skills, lower extremities, grip strength, cerebral palsy, balance

## Abstract

Background and objective

Cerebral palsy (CP) is one of the most prevalent neurological conditions affecting children; it is characterized by poor motor control, restricted range of motion (ROM), and poor balance. While whole-body vibration therapy (WBVT) has been used to treat these symptoms, its efficacy in different configurations remains unexplored. Hence, this study aimed to determine and compare the effects of WBVT applied to either the upper extremities, lower extremities, or both upper and lower extremities in weight-bearing and non-weight-bearing positions on ROM (shoulders, elbows, wrists, hips, knees, and ankle joints), balance, and function in children with spastic hemiplegic CP.

Methods

This randomized clinical trial involved 60 hemiplegic spastic CP children aged 5-15 years. After randomization, all the participants were divided into six groups of equal size based on the WBVT application for upper extremities, lower extremities, or both in weight-bearing or non-weight-bearing positions. The therapy was applied three times per week for four consecutive weeks. The outcome measures were ROM, hand grip strength, balance quantification score using My Fitness Trainer (MFT) 2.0, and timed up and go (TUG) scores.

Results

While all the groups were homogenous before treatment, after treatment, it was observed that all the ranges improved significantly in all groups. The same was observed for hand grip strength, balance score, and TUG test scores (p<0.05). The post-hoc analysis revealed that the weight-bearing position for the upper and lower extremities combined showed the highest level of improvement.

Conclusions

Based on our findings, WBVT in weight-bearing positions produces more significant results than in non-weight-bearing positions. We also observed that when WBVT is applied to the upper extremities, it can improve the function of the lower extremities and vice versa.

## Introduction

Cerebral palsy (CP) is one of the most common neurological conditions in children, and it is characterized by features such as motor disturbances, abnormal muscle tonicity, poor gait, poor balance, poor cognition, and behavioral issues. This condition is caused by injury to the developing brain during fetal life or after birth, which is a permanent and non-progressive one [1]. There are various ways to classify CP; the three most widely used classifications are spastic, flaccid, and athetoid. The spastic form of CP is the most prevalent variant. Another type of classification is based on the extremity involved, such as hemiplegic and diplegic. Sometimes, it can be classified as spastic hemiplegic, spastic monoplegic, etc [2]. It is more prevalent in preterm babies and those having delayed birth cry. The damage to the cerebral cortex is considered to be responsible for spasticity [3].

CP diagnosis is based on clinical assessment, usually in the first or second year of life. Various characteristic findings of CP include spasticity, poor coordination, cognitive issues, epilepsy, and perception and behavioral issues. Also, it is reported that CP children have poor proprioception [4]. The children often receive medication for managing spasticity, the effect of which is only short-lived and sometimes fruitless. Physical therapy is considered an integral part of patient management. The conventional physical therapy approach includes stretching the tight muscles, gait training, range of motion (ROM) exercises, and balance training [5]. In the past few years, whole-body vibration therapy (WBVT) has been proposed as a treatment modality for the management of CP. WBVT is a high-frequency, low-magnitude modality, with a vibrating platform that is extensively used to improve physical fitness. This modality is usually applied in a standing position on a vertical oscillating platform that changes the gravitational forces on the body and displaces the individual [6].

There are two types of WBVT machines: one produces side-to-side vibrations while the other produces sinusoidal vibrations. Sinusoidal vibration therapy is the most commonly used modality and is reported to be superior when compared to side-to-side vibration therapy [7]. WBVT induces reflex muscle activity via tonic vibration reflex (TVR). It has been reported that WBVT reduces muscle spasticity, improves gait speed and function of lower extremities, and induces relaxation [8]. During this therapy, the patient stands on the vibrating platform with slight knee flexion (i.e., weight-bearing position) [9]. This therapy is applied to the lower extremities, and there are no reports in the existing literature about the use of WBVT in the upper extremities in CP patients. Furthermore, a comparative analysis of this intervention in weight-bearing and non-weight-bearing configurations for both upper and lower extremities alone or altogether has not been performed yet.

As the vibratory information travels via the same pathways similar to proprioception ones, it is believed that WBVT can improve proprioceptive sense and thereby the overall function. The propriospinal tracts also play a significant role in the management of CP children. These tracts are considered to be responsible for interlimb coordination or neural coupling [10]. These vibrations have the potential to enhance neural drive to the muscles, and hence this augmented response enables the activation of motor units that are previously inactive, thereby increasing muscle mass and strength in CP [11].

Accordingly, we hypothesized that if we apply WBVT in weight-bearing and non-weight-bearing positions for either the upper or lower extremities alone or altogether, we may be able to observe a significant difference in patient outcomes. As weight-bearing positions produce more afferent information as compared to non-weight-bearing positions, we believe that weight-bearing positions will produce more significant changes than non-weight-bearing positions. We might also measure the effects of the interlimb coordination phenomenon by applying the therapy to the upper extremity and measuring its effects on the lower extremity and vice versa. We planned this study to test these hypotheses. We aimed to measure and compare the effects of WBVT applied in different configurations on the ROM, hand grip strength, balance quantification score, and timed up and go (TUG) test scores.

To sum up, the purpose of the present study was to determine and compare the effects of WBVT applied to either the upper extremities, lower extremities, or both upper and lower extremities in weight-bearing and non-weight-bearing positions on ROM, balance, and function in children with spastic hemiplegic CP. We believe the findings of this study will underscore the importance of adopting holistic approaches to therapy, targeting multiple limb domains to optimize functional outcomes and enhance patient well-being.

## Materials and methods

This randomized clinical trial was conducted as part of a research thesis for the degree of Ph.D. in Physical Therapy at Tehran University of Medical Sciences, Iran from May 2023 to February 2024. After obtaining ethical approval from the Research Ethics Committees of the School of Nursing and Midwifery & Rehabilitation-TUMS (IR.TUMS.FNM.REC.1402.024), the clinical trial was registered and approved by the Iranian Clinical Trial Registry (IRCT20090301001722N27) (https://irct.behdasht.gov.ir/trial/64165). The study was conducted at the Pakistan Airforce Base School for Persons with Special Needs (PAF PSNs) Nur Khan, Rawalpindi, Pakistan. It was a double-blinded study, with care providers blinded to the study groups (i.e., treatment and control groups), and outcome assessors blinded to the treatment protocols and study hypothesis. Two physical therapists, already working in the setting, participated in providing care and assessing outcomes. Each physical therapist assessed participants of the opposite gender in the presence of a schoolmaid. They then provided the treatment protocol, shared by the study's primary investigators, to participants of the same gender.

A total of 60 spastic CP children participated in the study (30 males and 30 females). All participants underwent screening to determine eligibility, the criteria for which were as follows: patients of both genders, aged 5-15 years, diagnosed with spastic CP, and having spasticity scores of 1, 1+, and 2 on the modified Ashworth scale, as well as Gross Motor Function Measure Level 1 and 2. Patients with any fixed musculoskeletal deformity, recent surgery (<1 year ago), unhealed fracture, epilepsy, auditory or visual problems, those receiving botulinum toxin therapy, those with other forms of CP (ataxic, flaccid, and dyskinetic), and patients with sensory loss in the upper and lower extremities were excluded. Written permissions were obtained from the parents of the children. We recruited 30 male and 30 female participants based on the inclusion criteria before randomization.

Randomization was then conducted separately for males and females, resulting in the formation of six groups (Groups Aa, Ab, Ac, Ba, Bb, and Bc), each comprising 10 participants. This approach ensured that each group contained an equal number of male and female participants. Randomization was conducted using Random Allocation Software (Version 1.0) developed by the Department of Anesthesia, Isfahan University of Medical Sciences, Isfahan, Iran. Participation in the study was voluntary. A timetable was created for the interventional phase of the study, and parents were requested to ensure the presence of participants during that time to prevent missed sessions. Parents/children who were not interested in finishing the course of treatments were also excluded.

All participants received treatments three days a week for four consecutive weeks, totaling 12 sessions. The treatment frequency for all groups was 18 Hertz, with an amplitude of 12 mm. The vibration frequency was increased by 1 Hertz per second until it reached 18 Hertz. Each session consisted of three minutes of vibration therapy followed by two minutes of rest, repeated four times during the 20-minute treatment. The groups received vibration therapy in different positions: Group Aa (WBVT in weight-bearing for upper extremities only was applied when the patients assumed a standing position and placed their both hands on the whole-body vibration platform, such that their elbow joints were slightly flexed; Group Ab (WBVT in weight-bearing for lower extremities only); Group Ac (WBVT in weight-bearing for both upper and lower extremities simultaneously); Group Ba (WBVT in non-weight-bearing for upper extremities only); Group Bb (WBVT in non-weight-bearing for lower extremities only), and Group Bc (WBVT in non-weight-bearing for both upper and lower extremities simultaneously). Additionally, all participants received conventional physical therapy, including stretching exercises, parallel bar exercises, and wobble board exercises for 15 minutes per day, as they had received before the study.

The outcome measures included joint ROM measured using a universal goniometer (shoulders, elbows, knees, and ankles), hand grip strength gauged using a Camry digital handheld dynamometer, balance quantification measured using MFT (My Fitness Trainer) 2.0 Disc, and TUG test. All participants were assessed before the intervention and immediately after undergoing the 12 sessions of WBVT therapy. There were no dropouts in the study. The ROM of the both upper and lower extremity joints were checked with a universal goniometer. The following joint ROMs were measured: shoulder joint, elbow joint, wrist joint, hip joint, knee joint, and ankle joint bilaterally. The test and retest reliability of a universal goniometer: intraclass correlation coefficient between 79%-92% [12]. ROM was measured using a universal goniometer without any zero error.

A digital hand-held dynamometer (Camry) was used to measure hand grip strength (kg). Before taking any measurements, we explained the procedure to all participants to ensure that they fully understood how to use the dynamometer. A demonstration of the grip test was provided, and participants were allowed to ask questions to clarify any uncertainties. Each participant performed one trial run on both the right and left hand to familiarize themselves with the device and the procedure. Participants were seated comfortably on a chair with their feet resting on the ground. The elbows were placed in a mid-flexed position (approximately 90 °). The dynamometer's handle opening was adjusted by the therapist for each participant to ensure an optimal and comfortable grip. Participants were instructed to squeeze the dynamometer maximally for three seconds. They were reminded to apply maximum effort during each squeeze. Three measurements were taken for each hand, with short rest periods between each squeeze to prevent muscle fatigue. The highest value from the three attempts was recorded as the hand grip strength for each hand. The test and retest reliability of digital hand-held dynamometer: intraclass correlation coefficient of 80%-94% [13]. Hand grip strength was measured using a digital hand-held dynamometer (Camry), which automatically sets to zero kilograms upon start-up. To ensure accuracy and reliability, we calibrated the dynamometer by attaching a known weight of 5 kg to its handle and verifying the measurement. As the dynamometer is a prefabricated tool, manual adjustments are not possible.

**Figure 1:**
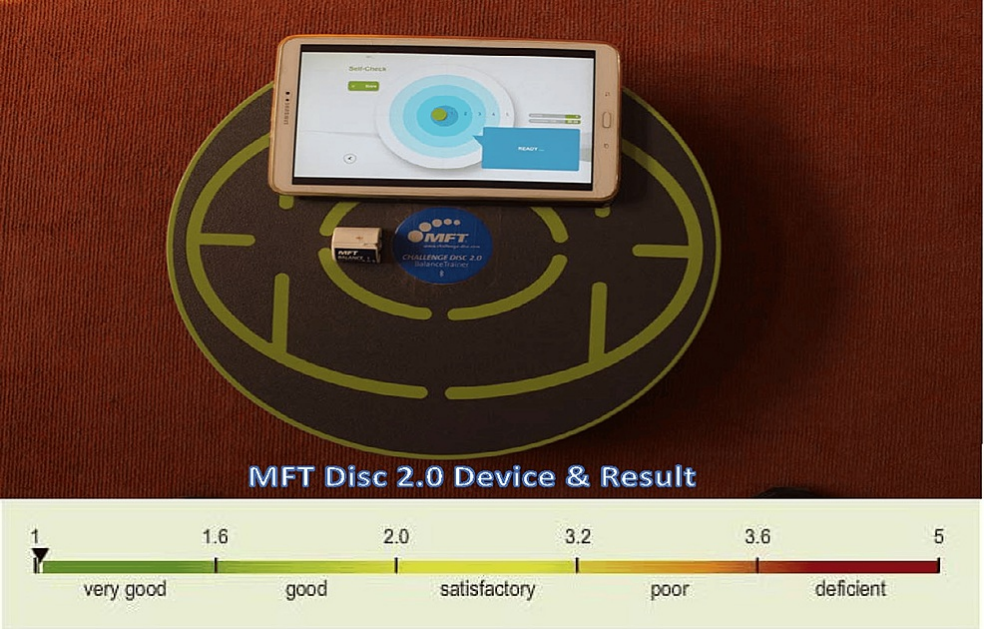
MFT Disc 2.0 - device and results MFT: My Fitness Trainer

MFT 2.0 disc is an electronic balance board used to quantify balance. It is also used for rehabilitation purposes. It is a Bluetooth-enabled device that can provide real-time visual feedback to the standing subjects as well. It has been used for the assessment and rehabilitation of skiers, athletes, gymnasts, and the geriatric population. The test system consists of a wooden platform with an attachable sensor, which measures all the deviations in the horizontal plane. All functions of the COG are recorded and transformed to define the state of balance. The result is based on stability and it can range from 1 to 5, as shown in Figure 1. All the procedures were explained and demonstrated until the participants understood them, and one practice session was conducted before the actual readings were taken. Test-retest reliability of balance quantification using MFT 2.0: intraclass correlation coefficient ranges from 68% to 81% [14]. MFT requires mandatory calibration at startup or before any test. The tool automatically calibrates itself within three seconds, provided it is kept balanced. This self-calibration process ensures the accuracy and reliability of the data collected by zeroing the instrument and preparing it for precise measurements.

The TUG test was used to assess the dynamic balance. All the participants were explained and demonstrated the procedure until they understood it and one practice session was conducted before the actual readings were taken. An armchair was placed 10 feet away from the wall, the participants had to stand up from the chair, walk the path, touch the wall, return to the chair, and sit back. The time was recorded using a digital stopwatch. Test-retest reliability of TUG test: intraclass correlation coefficient ranges from 87% to 99% [15]. TUG time was measured using a digital stopwatch.

Statistical analysis

The demographic data were presented using mean and standard deviation (SD). The normality of the data was checked and appropriate statistical tests were applied. As the data was normally distributed, one-way ANOVA was used on the pre- and post-treatment values. One-way ANOVA on pre-treatment values was used to test the homogeneity of the groups before treatment, while the same test on post-treatment values was used to analyze the group difference; the post-hoc test was applied to find the most effective group; a confidence interval of 95% and a level of significance of 5% was used. SPSS Statistics version 27 (IBM Corp., Armonk, NY) was used for data analysis.

## Results

A total of 60 patients were recruited in this study and divided into six groups of 10 participants each. The mean age in Group Aa was 11.80 ± 1.75, that in Group Ab was 11.90 ± 1.52 years, Group Ac was 12 ± 1.88, Group Ba was 11.60 ± 1.83, Group Bb was 12.10 ± 1.66 and Group Bc was 12.30 ± 1.88 years. All groups had an equal number of male and female participants (five each). While 41 participants had their right side affected, only 19 had their left side affected. One-way ANOVA showed no significant difference in age among the groups (p=0.965) (Table 1).

**Table 1:** Gender and mean age of the participants SD: standard deviation

Group (n)	Male (n)	Female (n)	Mean age in years	SD	P-value	Affected side	
						Right	Left
Aa (10)	5	5	11.80	1.75	0.965	9	1
Ab (10)	5	5	11.90	1.52		7	3
Ac (10)	5	5	12	1.88		5	5
Ba (10)	5	5	11.60	1.83		6	4
Bb (10)	5	5	12.10	1.66		6	4
Bc (10)	5	5	12.30	1.88		8	2

Table 2 shows the inter-group comparison of ROM. The pre-treatment ANOVA p-value for shoulder flexion ROM was 0.245, suggesting no significant difference between the groups before treatment. Post-treatment ANOVA p-value for shoulder flexion ROM was 0.000, indicating a statistically significant difference between the groups after treatment. The pre-treatment ANOVA p-value for shoulder extension was 0.667, meaning there was no significant difference between the groups before treatment. Post-treatment ANOVA p-value for shoulder extension ROM was 0.000, indicating a statistically significant difference between the groups after treatment.

**Table 2:** Inter-group comparison of range of motion ^*^Shows significant difference between groups in the post-hoc test SD: standard deviation

Joint/range in degrees/group				Aa	Ab	Ac	Ba	Bb	Bc	P-value
Shoulder	Flexion	Pre	Mean ± SD	111.2 ± 10.23	105.5 ± 5.31	114.2 ± 8.6	111.5 ± 8.64	113.9 ± 8.29	111.1 ± 8.58	0.245
		Post	Mean ± SD	129.3 ± 11.20	117.6 ± 6.75	138.7 ± 6.01*	121.1 ± 7.32	120.5 ± 8.65	125.1 ± 8.9	0.000
	Extension	Pre	Mean ± SD	34 ± 5.88	36 ± 5.20	35.1 ± 5.62*	33.5 ± 5.72	34.8 ± 4.94	31.9 ± 6.33	0.667
		Post	Mean ± SD	46.1 ± 4.17	42.3 ± 4.99	52.5 ± 3.74*	41.1 ± 5.54	39.7 ± 4.78	42 ± 6.99	0.000
	Adduction	Pre	Mean ± SD	28 ± 6.61	28.9 ± 4.86	30 ± 4.44*	29 ± 6.73	27.9 ± 6.95	30.9 ± 5.46	0.857
		Post	Mean ± SD	37.7 ± 6.25	35.8 ± 4.61	43.4 ± 4.97*	37 ± 5.73	33.2 ± 6.63	39.9 ± 5.99	0.006
	Abduction	Pre	Mean ± SD	74.5 ± 6.88	75.2 ± 5.26	75.7 ± 4.83*	74.6 ± 6.75	73.8 ± 6.95	76.4 ± 6.18	0.950
		Post	Mean ± SD	91.4 ± 8.00	81.5 ± 5.29	102.3 ±10.37	83.7 ± 6.75	78.8 ± 8.99	88.5 ± 5.81	0.000
	Internal rotation	Pre	Mean ± SD	60 ± 9.58	62.1 ± 5.99	60.9 ± 10.27	63 ± 8.98	65 ± 10.79	64 ± 11.48	0.862
		Post	Mean ± SD	69.7 ± 8.44	67.4 ± 5.89	77.2 ± 9.24	69 ± 8.55	69.2 ± 10.84	71.2 ± 12.36	0.264
	External rotation	Pre	Mean ± SD	44.9 ± 12.27	50.2 ± 13.89	48 ± 11.96	50.9 ± 11.41	54.6 ± 7.63	48.9 ± 11.62	0.574
		Post	Mean ± SD	56 ± 12.71	56.1 ± 13.64	63.1 ± 10.65	57.2 ± 11.30	58.4 ± 7.38	58.9 ± 10.70	0.737
Elbow	Flexion	Pre	Mean ± SD	95.1 ± 15.95	98 ± 18.80	99.7 ± 17.30	100.9 ± 14.32	103.9 ± 16.80	102.9 ± 17.27	0.866
		Post	Mean ± SD	110.4 ± 16.00	105.2 ± 17.76	129.7 ± 8.55*	112.4 ± 14.72	110.1 ± 15.75	117.6 ± 16.21	0.013
	Extension	Pre	Mean ± SD	-13.9 ± 6.33	-12 ± 6.91	-15 ± 7.02	-17.9 ± 7.37	-12.9 ± 5.68	-11.1 ± 7.82	0.301
		Post	Mean ± SD	-6.8 ± 5.86	-9.2 ± 7.02	-4.7 ± 3.83*	-13.6 ± 9.39	-10.1 ± 5.25	-5.9 ± 5.34	0.034
Knee	Flexion	Pre	Mean ± SD	65.8 ± 15.33	70.1 ± 18.33	63.3 ± 14.87	68.9 ± 19.99	67 ± 18.23	75.3 ± 14.57	0.707
		Post	Mean ± SD	69.6 ± 14.87	78.2 ± 18.06	76.7 ± 14.09*	71.8 ± 20.19	71.4 ± 17.96	80.5 ± 14.64	0.047
	Extension	Pre	Mean ± SD	-11 ± 4.61	-10.6 ± 3.13	-10.8 ± 3.91	-11.3 ± 3.97	-11.5 ± 2.95	-11.7 ± 3.12	0.984
		Post	Mean ± SD	-9.4 ± 3.89	-6.2 ± 2.57	-5.1 ± 3.66*	-10.6 ± 3.68	-9.3 ± 2.62	-8 ± 2.78	0.003
Ankle	Plantar flexion	Pre	Mean ± SD	34.9 ± 5.32	36 ± 4.37	34.8 ± 6.21	38.1 ± 4.53	36 ± 5.49	36.5 ± 5.62	0.760
		Post	Mean ± SD	36.4 ± 5.4	42.3 ± 4.34	48.3 ± 6.43*	39.5 ± 4.64	39.9 ± 5.54	41.3 ± 5.18	0.000
	Dorsiflexion	Pre	Mean ± SD	7 ± 2.58	7.9 ± 3.44	9 ± 2.62	7.5 ± 2.87	8.5 ± 3.27	7.9 ± 3.28	0.740
		Post	Mean ± SD	9.1 ± 2.76	13.5 ± 3.97	19.3 ± 2.00*	8.9 ± 3.41	11.6 ± 3.16	12.2 ± 2.97	0.000

The pre-treatment ANOVA p-value for shoulder adduction was 0.857, meaning there was no significant difference between the groups before treatment. Post-treatment ANOVA p-value for shoulder adduction was 0.006, indicating a statistically significant difference between the groups after treatment. Pre-treatment ANOVA p-value for shoulder abduction was 0.950, meaning there was no significant difference between the groups before treatment. Post-treatment ANOVA p-value for shoulder abduction was 0.000, meaning there was a statistically significant difference between the groups after treatment.

The pre-treatment ANOVA p-value for the shoulder internal rotation ROM was 0.862, meaning there was no significant difference between the groups before treatment. Post-treatment ANOVA p-value for the shoulder internal rotation ROM was 0.264, meaning there was no significant difference between the groups after treatment. The pre-treatment ANOVA p-value for the shoulder external rotation ROM was 0.574, meaning there was no significant difference between the groups before treatment. Post-treatment ANOVA p-value for shoulder external rotation ROM was 0.737, meaning there was no significant difference between the groups after treatment.

The pre-treatment ANOVA p-value for elbow flexion was 0.866, meaning there was no significant difference between the groups before treatment. Post-treatment ANOVA p-value for elbow flexion was 0.013, meaning there was a statistically significant difference between the groups after treatment. For elbow extension, the pre-treatment ANOVA p-value was 0.301, meaning there was no significant difference between the groups before treatment. Post-treatment ANOVA p-value for elbow extension was 0.034, meaning there was a statistically significant difference between the groups after treatment.

For knee flexion ROM, the pre-treatment ANOVA p-value was 0.707, meaning there was no significant difference between the groups before treatment. Post-treatment ANOVA p-value for knee flexion ROM was 0.047, meaning there was a statistically significant difference between the groups after treatment. For knee extension ROM, the pre-treatment ANOVA p-value was 0.984, meaning there was no significant difference between the groups before treatment. Post-treatment ANOVA p-value for knee extension ROM was 0.003, meaning there was a statistically significant difference between the groups after treatment.

The pre-treatment ANOVA p-value for ankle plantar flexion ROM was 0.760, indicating no significant difference between the groups before treatment. The post-treatment ANOVA p-value for ankle plantar flexion was 0.000, meaning there was a statistically significant difference between the groups after treatment. The pre-treatment ANOVA p-value for ankle dorsiflexion ROM was 0.740, meaning there was no significant difference between the groups before treatment. The post-treatment ANOVA p-value for ankle dorsiflexion ROM was 0.000, meaning there was a statistically significant difference between the groups after treatment. The post-hoc analysis revealed that all the ranges in Group Ac improved more significantly than other groups. 

The pre-treatment ANOVA p-value for hand grip strength was 0.985, indicating that there was statistically no significant difference between the groups. The one-way ANOVA p-value for post-treatment was 0.000, meaning a statistically significant difference between the groups. The post-hoc analysis revealed that Group Ac improved more significantly than other groups (Table 3).

**Table 3:** Inter-group comparison of hand grip strength ^*^Shows significant difference between groups in the post-hoc test SD: standard deviation

Group	Pre-treatment values in KG			Post-treatment values in KG		
	Mean	SD	P-value	Mean	SD	P-value
Aa	8.14	1.19	0.985	13.17	1.19	0.000
Ab	8.57	2.56		11.33	2.35	
Ac	8.33	2.23		15.53*	2.09	
Ba	8.22	1.92		11.94	1.68	
Bb	8.79	2.07		11.27	1.78	
Bc	8.49	2.40		14.21	2.36	

The one-way ANOVA p-value for the pre-treatment MFT score was 0.932, meaning that there was statistically no significant difference between the groups. ANOVA p-value for the post-treatment MFT score was 0.000 meaning that there was statistically no significant difference between the groups. The post-hoc analysis revealed that Group Ac improved more significantly than other groups (Table 4).

**Table 4:** Inter-group comparison of MFT Disc 2.0 scores ^*^Shows significant difference between groups in the post-hoc test MFT: My Fitness Trainer; SD: standard deviation

Group	Pre-treatment values			Post-treatment values		
	Mean	SD	P-value	Mean	SD	P-value
Aa	4.5	0.21	0.932	3.9	0.17	0.000
Ab	4.6	0.23		3.5	0.97	
Ac	4.6	0.27		3.1*	0.08	
Ba	4.5	0.18		3.9	0.17	
Bb	4.5	0.16		3.8	0.22	
Bc	4.5	0.2		3.2	0.13	

The one-way ANOVA p-value for the pre-treatment TUG score was 0.053, meaning that there was statistically no significant difference between the groups. The ANOVA p-value for the post-treatment TUG score was 0.000, meaning that there was statistically no significant difference between the groups. The post-hoc analysis revealed that Group Ac improved more significantly than other groups (Table 5).

**Table 5:** Inter-group comparison of timed up and go test ^*^Shows significant difference between groups on the post-hoc test SD: standard deviation

Group	Pre-treatment values in seconds			Post-treatment values in seconds		
	Mean	SD	P-value	Mean	SD	P-value
Aa	14.90	4.84	0.053	12.03	4.00	0.000
Ab	12.60	3.28		8.58	1.83	
Ac	14.71	4.28		8.33*	1.86	
Ba	14.64	3.92		12.01	3.03	
Bb	13.08	3.16		9.50	1.65	
Bc	17.86	2.99		12.53	2.19	

## Discussion

The results of our study demonstrated notable variations in several key parameters among different treatment groups, including ROM, hand grip strength, TUG test scores, and the MFT score. By utilizing ANOVA, we found that all groups exhibited homogeneity at the pre-treatment stage. Hence, these findings reflect the normal distribution of data in the participants. Besides, the results showed statistically significant differences in post-treatment assessment. Interestingly, Group Ac consistently showed the most significant improvements across various variables, indicating the efficacy of this treatment approach.

Regarding ROM, our findings suggested that WBVT applied in weight-bearing positions for both upper and lower extremities simultaneously yields substantial improvements, which aligns with previous research by Ahmadizadeh et al. (2019), who reported enhanced ROM in CP children following a similar intervention [16]. Additionally, our results indicated a positive effect of WBVT on hand grip strength, in line with studies by Ahn et al. (2018) and de Oliveira R et al. (2023), although the latter suggested that the effect may be limited to lower extremity strength [17].

Furthermore, the significant improvement in TUG scores post-treatment points to the potential of WBVT to enhance functional mobility, consistent with findings by Cheng et al. (2015) [18]. The results by Pin et al. (2019) showed no improvement in TUG scores, which is not in line with the bulk of results provided by other research, including the present study. Hence, we suggest that the differences in their study could be attributed to the variation of GMFM levels (III and IV), and the utilization of 2 mm amplitude [19]. We have noted a significant improvement in balance test scores measured by MFT Disc 2.0, corroborating findings by various researchers such as Parashar et al. (2017) [20], Son et al. (2019) [21], and Jung et al. (2020), indicating the potential of WBVT to address stability and balance deficits in CP children [22].

Our findings have demonstrated that WBVT applied to either the upper or lower extremities, in weight-bearing or non-weight-bearing positions, enhances functional outcomes across limb domains. This phenomenon could be attributed to mechanisms such as cross-training effects, neural coupling, or interlimb coordination, as suggested by Weersink et al. (2022). They proposed enhanced bidirectional coupling between shoulder and leg muscles at subcortical and transcortical levels in Parkinson's patients, potentially compensating for reduced common cortical driving sources [23]. Toth et al. (2022) proposed that sensory inputs from the hand rapidly establish connections with leg motor neurons via spinal pathways, facilitating rapid adjustments in balance responses [24]. Sidiropoulos et al. (2019) have demonstrated that intensive upper extremity interventions positively impact spatiotemporal coordination between arms and legs in children with CP [25].

Ferris et al. (2006) have suggested that active stepping with increased arm resistance led to coordinated leg muscle activity and reciprocal muscle activation, indicating the potential for upper extremity interventions to influence leg movements [26]. Dietz (2002) has proposed flexible coupling between thoracolumbar and cervical centers, allowing for task-dependent gating of neuronal pathways between upper and lower limb muscles during locomotion, as evidenced by arm swing's residual function from quadrupedal locomotion [27]. Green and Gabriel (2018) have described the rehabilitative benefits of cross-education, suggesting strength gains and prevention of strength loss through unilateral training, possibly via the cross-activation or bilateral access theories [28]. Kassem (2020) and El-Hadidy (2004) further supported cross-education's potential in improving functional recovery and upper extremity rehabilitation in CP patients [29,30].

Our study highlights the significant role of upper extremities WBVT in lower extremities function and performances, particularly in patients who are unable to engage in lower limb WBVT. Besides, we delve into the effects of lower limb WBVT exercises on upper limb function, shedding light on the potential reciprocal benefits for limbs in rehabilitation interventions for conditions like CP. These findings underscore the importance of adopting a holistic approach to therapy, targeting multiple limb domains to optimize functional outcomes and enhance patient well-being.

This study has a few limitations. It focused solely on hemiplegic spastic CP children aged 5-15 years, and hence its findings may not be generalizable to other types of CP or patients of different age groups. Additionally, the follow-up period in our study was only four weeks, which might not be sufficient to capture the long-term effects of WBVT.

## Conclusions

Based on the findings of our study and the extensive review of the literature, WBVT holds significant promise in improving various parameters related to the upper and lower extremity function and performance. Our results indicate that WBVT, whether applied to the upper or lower extremities in either weight-bearing or non-weight-bearing positions, leads to substantial improvements in ROM, hand grip strength, mobility, and balance. The effectiveness of WBVT may be attributed to mechanisms such as cross-training effects, neural coupling, and interlimb coordination, as elucidated by previous research. These mechanisms highlight the intricate connections between upper and lower limb muscles, underscoring the potential for targeted interventions to yield comprehensive improvements across limb domains.

Furthermore, our study underscores the importance of adopting a holistic approach to therapy, particularly in individuals with limited mobility who may benefit from upper extremity WBVT when lower extremity exercises are not feasible. Additionally, the reciprocal benefits of lower limb WBVT on upper limb function further emphasize the interconnectedness of limb domains in rehabilitation interventions. Our findings add to the growing body of evidence supporting the efficacy of WBVT in enhancing functional outcomes in individuals with various neuromuscular conditions. Going forward, it is imperative to continue to explore the nuanced effects of WBVT and develop tailored intervention strategies to optimize outcomes and improve overall patient well-being.
